# Indications for the Use of Nitro-Glycerine

**Published:** 1887-12

**Authors:** 


### *INDICATIONS FOR THE USE OF NITRO-GLYCERINE.*

The value of nitro-glycerine in various diseases, as angina pectoris, hemicrania, and neuralgia, and also in sea-sickness, certain forms of anæmia, etc., depends on the existence in these of an irregular distribution of the blood. This abnormal condition may be recognized by a certain grade of pallor of the skin, especially of the face, an appearance co-existent with a weak pulse and small radial arteries, hard and frequently situated at a certain depth. When, on the contrary, the headache and neuralgia occur in persons with chronic congestion of the subcutaneous vessels of the face, nitro-glycerine is contra-indicated; and similarly it should not be used in asthma when the face is congested from the effects of the emphysema. Thus it may be said that the best therapeutic results from nitro-glycerine may be obtained in those cases in which angina pectoris, neuralgia, etc., are associated with pallor of the countenance.

The c ondition of the pulse is the best indication for the use of nitro-glycerine, and the safest guide for the determination of the time in which one should begin the cure. The smaller the radial artery is, so much the more rapidly does it dilate under the influence of the drug, and so much the less are the secondary effects produced by it; on the contrary, the fuller the pulse, and the more tense the radial artery, so much the less this resents the influence of it.

When the pulse is small, the usual dose of one drop of a one per cent. solution is sufficient, while if the pulse is large, two drops may be required to obtain the full effect. When the radial is soft and the pulse weak, smaller doses should be given—one-half to one-fourth of a drop. The sensations experienced by the patient, throbbing and pain in the head, as well as the distension of the radial artery under the observer's finger, should be the guide for the increase of the dose.—*Giornale Internazionale delle Scienze Mediche.*

